# Microfluidic Chips: Emerging Technologies for Adoptive Cell Immunotherapy

**DOI:** 10.3390/mi14040877

**Published:** 2023-04-19

**Authors:** Yishen Tian, Rong Hu, Guangshi Du, Na Xu

**Affiliations:** 1 Translational Medicine Research Center, Guizhou Medical University, Guiyang 550025, China; 2 Institute of Biology and Medicine, College of Life Sciences and Health, Wuhan University of Science and Technology, Wuhan 430065, China

**Keywords:** microfluidic chip, adoptive cell therapy, cell sorting, cell expanding, immunotherapy

## Abstract

Adoptive cell therapy (ACT) is a personalized therapy that has shown great success in treating hematologic malignancies in clinic, and has also demonstrated potential applications for solid tumors. The process of ACT involves multiple steps, including the separation of desired cells from patient tissues, cell engineering by virus vector systems, and infusion back into patients after strict tests to guarantee the quality and safety of the products. ACT is an innovative medicine in development; however, the multi-step method is time-consuming and costly, and the preparation of the targeted adoptive cells remains a challenge. Microfluidic chips are a novel platform with the advantages of manipulating fluid in micro/nano scales, and have been developed for various biological research applications as well as ACT. The use of microfluidics to isolate, screen, and incubate cells in vitro has the advantages of high throughput, low cell damage, and fast amplification rates, which can greatly simplify ACT preparation steps and reduce costs. Moreover, the customizable microfluidic chips fit the personalized demands of ACT. In this mini-review, we describe the advantages and applications of microfluidic chips for cell sorting, cell screening, and cell culture in ACT compared to other existing methods. Finally, we discuss the challenges and potential outcomes of future microfluidics-related work in ACT.

## 1. Introduction

The immune system plays a crucial role in controlling tumor growth. In recent years, immunotherapy has emerged as a powerful potential treatment for various types of cancer [1,2]. Adoptive cell therapy (ACT) is a rapidly developing field of cancer immunotherapy that had been active since the 1980s [3]. A landmark in cancer immunotherapy occurred in 2017, when the US Food and Drug Administration (FDA) authorized two chimeric antigen receptor T cells therapies (CAR-T) (Kymriah and Yescarta) for the treatment of B-cell malignancies in children and adults. These treatments received approval soon afterwards in the EU, UK, and Canada in 2018 [4]. Currently, six kinds of CAR-T medicines have received FDA approval for the treatment of some hematologic malignancies, including B-cell lymphoma and bone marrow tumors. Numerous ACT medicines are now in the phase of clinical testing.

Currently, T cell-based ACT can be divided into three different types with different mechanisms of action: TIL, T-cell receptor-engineered T cell therapies (TCR-T) and CAR-T. The use of other immune cell types, such as natural killer cells, as the basis for ACT is also an area of current research [4,5]. The in vitro processes of PBMC isolation, genetic engineering modification, and cell expansion are pivotal for a successful treatment. In the case of TIL treatment, for example, a patient required the removal of one or more tumors with a total diameter of at least 2–3 cm in a treatment plan for metastatic melanoma [6]. The excised tumor was fragmented or enzymatically digested, and subsequently, TILs were isolated and cultured in the presence of interleukin-2 (IL-2) to promote TIL proliferation. The initial growth phase took approximately 14 days. When the CD3^+^ T cells reached high enough numbers, their anti-tumor specificity was tested by quantifying the production of interferon-γ (IFN-γ) in the presence of an autologous tumor cell line. T cells with anti-tumor specificity were isolated, re-expanded, and injected into patients who had already undergone lymphocyte depletion therapy. Simultaneously, IL-2 was administered after T-cell infusion to facilitate engraftment and prolong the lifespan of the injected T-cells [7]. For TCR-T and CAR-T, massive isolation of highly active T cells from the blood is required, followed by transduction of the TCR or CAR gene using a retrovirus, resulting in high levels of expression of the introduced TCR/CAR. The cells were then expanded in vitro to reach the number of cells required for treatment [8]. Before moving on to the subsequent manufacturing stage, each of the aforementioned steps in the process must pass rigorous testing and comply with requirements [9]. Moreover, the development and production of ACT is moving towards personalized therapeutics, and each product needs a separate GMP-grade space for production [10]. Additionally, the majority of T cell and NK cell generation currently takes place in static culture bags, which is inefficient for amplification [11].

Microfluidic chips have become increasingly popular in research domains including biochemical analysis and drug screening due to their advantages of integration, high efficiency, and low sample demand [12,13]. Microfluidic chips are used in processes such as cell separation, culture, and detection [14,15]. The desired immune cells can be automatically and effectively isolated from the blood of a patient using microfluidic cell separation techniques based on mechanical, chemical, electrical, and magnetic principles [16,17]. The small size of the microfluidic chip microstructure can also mimic the native microenvironment of cells better, which facilitates the in vitro culture of immune cells [18,19]. Therefore, microfluidics chips offer various special benefits in the ACT field. The first benefit is the integration and automation of microfluidic chips. Currently, a course of treatment using CAR-T products costs between USD 300,000 and 500,000. The high cost of production is due to the necessity of rigorous testing at each stage of CAR-T production, which is one reason for the treatment expense. However, costs could be significantly reduced by creating integrated microfluidic chips that combine the production steps of T-cell sorting, modification, and proliferation. By using microfluidics, it is conservatively estimated that the cost of ACT can be reduced to one-seventh of the current cost. Another advantage of microfluidic chips is their ideal suitability to personalized requirements, which are now being implemented in ACT [20]. Personalized treatment requires each product to be produced in a separate Good Manufacturing Practice of Medical Products (GMP) facility with a single incubator and ventilation system that can only be used to culture the cells of a single patient. For the facility, there are very stringent space requirements. The challenge of space can be resolved using microfluidics, as personalized products do not need to be produced in large volumes. Finally, the high-throughput advantage of microfluidics allows for the exploration of conditions for multiple factors in the drug development process, especially when immune cells are expanded in vitro. Microfluidics allow for the exploration of the effects of multiple factors such as three-dimensional culture, co-stimulatory factor type and concentration, and oxygen content on immune cell expansion, thereby accelerating the screening of medicine candidates. In summary, the microfluidic chip is expected to solve key problems in the development and production of ACT, and ACT may become a key application in the microfluidic chip industry.

**Scheme 1:**
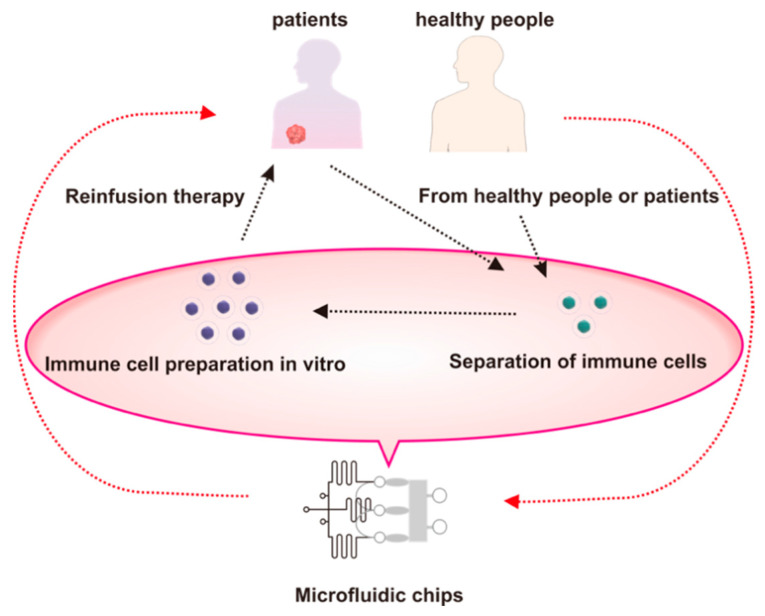
Schematic illustration of a microfluidic chip in adoptive cell therapy. Microfluidic chips can combine cell isolation and culture into a single integrated chip, which realizes the automatic and personalized production of target cells and supplies a novel solution for high-cost adoptive cell therapies (ACT).

With the continuous development of different microfluidic technologies, the application of microfluidics in ACT has received great attention. It is essential to have a thorough understanding of microfluidic techniques suitable for ACT treatment, particularly the advantages and limitations of these techniques in ACT. The strategy in ACT is complex and variable, and both cell isolation and in vitro cell culture are limited by current technology, resulting in a cumbersome and time-consuming process. The activity and function of the final cells obtained may be compromised, thus limiting the therapeutic effect of subsequent ACT. In this mini-review, the application of microfluidics to two important steps in ACT, the isolation of immune cells and the in vitro expansion of functional immune cells, is analyzed and discussed. Firstly, the achievements to date in the use of microfluidic platforms in cell isolation and cell culture studies are described. The feasibility and limitations of each of these achievements in ACT are also discussed. Secondly, two novel integrated chips combining microfluidic cell isolation and cell culture technologies are presented. The integrated microfluidic chip was used to create personalized anti-tumor cell agents and to automate their production (Scheme 1). The advantages of microfluidics, such as integration, automation, personalization, and high throughput, are well represented. Finally, the opportunities and challenges of microfluidics are prospected for future ACT research.

## 2. Microfluidic Cell Sorting Chip for ACT

Cell sorting is involved throughout the production process of ACT, including the separation of PBMC from whole blood or screening of target gene-modified TCR-T or CAR-T cells after viral transduction. Currently, flow cytometry, immunomagnetic bead sorting, density gradient centrifugation, and adhesion quality-based cell separation are common techniques for separating cells (Table 1). Each technique has its advantages and disadvantages. The density gradient centrifugation method may not be satisfactory to ensure the separation purity of some immune cells with gravity similar to lymphocytes. For flow cytometry, fluorescent markers or dyes are necessary. For immunomagnetic bead sorting, high-cost antibody-coated magnetic spheres must be added. These additions have a substantial impact on the following treatments [21,22].

The precise identification and separation of functional cells in microchannels has been accomplished by the design and manufacturing of micro-structured channels which use chemical/physical techniques, microfluidic principles, and other approaches [23]. Microfluidics have become more prominent in efficient cell separation techniques such as circulating tumor cell (CTCs) detection, single cell, and stem cell isolation [24]. According to cell separation principles, microfluidic cell separation methods [25] are currently classified as hydrodynamic force, acoustic, optical, etc.

### 2.1. Hydrodynamic Force-Driven Cell Separation

Hydrodynamics are the basic and inherent physical principles for the implementation of microfluidic systems. Based on the principles of hydrodynamics, additional principles and methods such as magnetic, acoustic, and optical tweezers can be combined to achieve specific functions. Dean flow and deterministic lateral displacement (DLD) are two common methods for separating cells using hydrodynamic forces [17,26]. The fundamental idea behind both approaches is based on the fact that cells of various sizes are subjected to various microfluidic forces in the designed microchannels [27,28].

**Figure 1:**
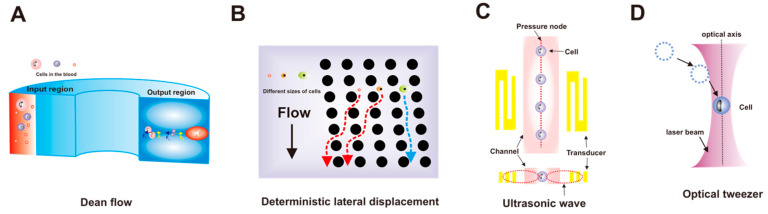
Schematic of cell separation principles. (**A**,**B**) Hydrodynamic forces act on the cells in the microchannel system. Reproduced with permission from Zhang et al., Lab Chip 21(22), 4414–4426 (2021). Copyright 2021 The Royal Society of Chemistry [26]. (**C**) The ultrasonic waves produced by transducers are used as the acoustic primary radiation force for acoustic cell separation. (**D**) The gradient forces of the laser beam are applied using optical tweezers. Reproduced with permission from Park et al., Journal of Micromechanics and Microengineering, 26(1), 013003 (2016). Copyright 2016 IOP Publishing [25].

In curved microchannels (Figure 1A), the resistance caused by the dean flow and the inertial lift caused by the flow profile (Saffman lift) cause the microparticles to settle in an equilibrium position. The dean drag force (F_D_) and inertial lift force (F_L_) can be described by the following Equations (1) and (2).

(1) $$F_{D}=3\pi \mu Ua_{p}$$

(2) $$F_{L}=f_{L}\rho U^{2}a_{p}^{4}/D_{h}^{2}$$

The formulas show that the dean drag force (F_D_) and inertial lift force (F_L_) are proportional to $a_{p}$ and $a_{p}^{4}$, respectively, where $a_{p}$ is the particle diameter [29]. Thus, particles of different sizes can be separated by resistance or lift forces. Using these mechanisms, size-based separation studies have been performed on scales of up to 1–3 μm [29]. Separation of blood cells has also been demonstrated [30]. For example, Lee et al. used non-diluted whole blood samples with the addition of various human breast cancer cells, such as MCF-7, SK-BR-3, and HCC70, and achieved a cancer cell separation rate of 99.1%, blood cell rejection ratio of 88.9%, and throughput of 1.1 × 10^8^ cells min^−1^ [25]. In another study, large-sized cells, such as monocytes, were mainly affected by inertial lift and migrated toward the inner wall, while small-sized cells, such as erythrocytes and lymphocytes, were mainly affected by dean flow and migrated towards the outer wall. Immune cells in the blood can be separated through repetitive dean flow fields generated in continuously contracting and expanding microchannels [31].

DLD is another method of cell separation using hydrodynamic and channel geometry force balance (including fluid resistance from array columns). The fundamental concept is as follows: different drag forces and DLD forces are applied to the cells as they move along the streamlines of the flow, depending on the diameter of the cell. If cells are driven into neighboring streams by interactions with posts (Figure 1B), their overall trajectories are altered, and separation depends on whether or not they can follow a single lamina in the general direction of the fluid flow. Using this mechanism, different types of cells such as lymphocytes (8–12 μm), monocytes (15–20 μm), red blood cells (6–8 μm), and even circulating tumor cells (CTC) can be isolated from the blood [15,17].

The advantages of the above methods are that they cause less physiological damage to cells during isolation, higher throughput, are convenient and rapid, require low personnel and instrumentation, and do not require the addition of other substances (e.g., fluorescent antibodies, immune-encapsulated magnetic beads, etc.) which have little effect on subsequent cell incubation. However, the separation purity is weak, and chips are prone to blockages. Moreover, in ACT therapy, specific cell subpopulations need to be isolated, for example, CD4^+^ T cells, which need to be separated from CD8^+^ T cells, which are similar in cell size. Therefore, this method is more difficult to precisely isolate functional cells. Additional equipment can be added to improve the specificity of the separation, such as immunomagnetic beads.

### 2.2. Acoustic Force-Driven Cell Separation

A non-contact technique for sorting cells is the acoustic approach, which makes use of ultrasonic standing waves [32]. In most microfluidic systems that use a liquid medium as the working fluid, the particles in the liquid medium are subjected to the acoustic primary radiation force (the maximum force on the particles associated with harmonic standing waves) and secondary acoustic force (the secondary force generated by the waves scattered by the particles). Therefore, the use of pressure gradients produced by ultrasonic standing waves is the core tenet of acoustic sorting [33]. The pressure gradient exerts a force on the particle, and when the acoustic contrast factor is positive, the particle moves to the pressure node (where the pressure change is zero). Conversely, when the acoustic contrast factor is negative, the particle moves to the pressure backline (where the maximum pressure change occurs). Different bioparticles (cells, proteins, etc.) have different acoustic contrast factors [34]. Thus, a combination of different acoustic devices and microfluidic channels can be used for high-throughput isolation of cells. For example, in reference [35], the piezo ceramic actuator was adhered between the side walls of a microfluidic channel, creating pressure nodes in the channel. As the cells advanced along the channel, they were translated by acoustic forces to the center of the channel. The purity of this separation was determined by the size, density, compressibility, and acoustic pressure amplitude of the cells (Figure 1C). In 2012, Ding et al. showed how to use programmable standing-surface acoustic waves (SSAW) to create a microfluidic device for continuous flow cell sorting [36]. The floating leukemia cells were quickly sorted to the specified outlets through the controlled or determined pressure nodes.

This technique for separating immune cells has the benefit of high throughput and reduced clogging. However, after isolation, the cells in the ACT must remain highly active, as gene editing or cell proliferation will subsequently be required in vitro. It is currently unknown whether the acoustic isolation of immune cells affects the results of later cell manipulation. In addition, whether this approach can isolate more specific functional cells needs further exploration.

### 2.3. Optical Tweezer-Driven Cell Separation

Optical tweezer technology is based on optical radiation pressure and single-beam gradient force light traps. When light strikes an object, the electromagnetic wave has energy and momentum. The electromagnetic wave is reflected and absorbed on the surface of the object, creating pressure on the surface which becomes the optical pressure (or optical radiation pressure). The use of a tightly focused laser beam provides very high precision in the selection and manipulation of cells (Figure 1C). The laminar flow characteristics of microfluidics allow the targeted cells to become concentrated in a specific location. The laser beam and microchannels are combined to produce an optical tweezer microfluidic system. The target cells can be moved in a non-invasive and precise manner through the optical tweezers into the desired microfluidic channel, thus achieving high-precision cell separation. A universal single-cell manipulation optical tweezer microfluidic system for high accuracy sorting of small cell populations was introduced by Wang et al. [35]. Microfluidic channels were employed for the aggregation and inducement of target cells, and optical tweezers were guided by the CCD camera images to process and move the target cells to the desired exit. After cell sorting, a purity of 96% (5 × 10^7^ particles mL^−1^) of yeast cells and a purity of 90% (1 × 10^7^ particles mL^−1^) of human embryonic stem cells (hESC) was achieved.

The precise isolation of functionally distinct immune cells and their subpopulations, as well as the isolation of biomolecules, can all be achieved with optical tweezers because they provide greater accuracy (up to 10 nm) than conventional techniques. However, this method has a low throughput (<0.1 μL/min) and requires complicated equipment setup; therefore, further research and work are still necessary to apply this method to ACT.

### 2.4. Dielectrophoretic Force-Driven Cell Separation

The microfluidic cell separation chip based on dielectrophoresis (DEP) combines the advantages of DEP and microfluidic chip technology, making it a hot research topic both domestically and internationally. DEP cell separation is a label-free and rapid method that analyzes cells based on physical changes rather than chemical changes, which require the use of molecular markers. Various cells have different dielectric properties (conductivity, dielectric constant), which induce different degrees of polarization when subjected to non-uniform AC electric fields. During DEP-based chip separation, the cells are subjected to positive or negative DEP forces due to their different dielectric properties, which will move the cells to specific positions in the inhomogeneous electric field. This method can be used to identify cells with different dielectric properties, such as non-viable human T-cells which have significantly different dielectric properties than viable human T-cells [37]. The greatest advantage of DEP-based chip separation of cells is its high purity of separation. Ringwelski et al. used DEP-based chips to separate cell samples and achieved 100% purity and high viability of isolated cells (>90%) [38].

For this method to be used in ACT treatment, it is necessary to further investigate whether the temperature change generated by the electric field has any effect on the physiological state of the cells, especially the subsequent cell proliferation and differentiation.

## 3. Microfluidic Cell Screening for ACT

Cell screening, especially single cell screening, is crucial for ACT. For example, in the CAR-T development process, there is a need to screen out highly transactive, highly lethal CAR sequences. After screening out the cells expressing CARs, the CAR-Ts are amplified, and their killing effect is tested. It is common to use 96-well plates in which an infinitely diluted cell suspension is spread into the wells, thus enabling single-cell culture from which high CAR-expressing T cells are subsequently sought. Traditional methods of cell screening require multiple platforms to work together, leading to high manual demands and tedious work.

Droplet microfluidic systems (DMSs) have become an indispensable tool for performing single-cell screening assays [39,40]. High-throughput antibody screening utilizing DMSs is now the common method for cell screening [41]. A DMS allows cells to be analyzed and screened at the single-cell level at high throughputs, which is not possible using traditional analytical methods. The DMS can encapsulate individual cells in water-in-oil droplets at a rate of thousands of droplets per second. Antibodies produced by the cells are contained within the droplet, allowing the phenotype and genotype within the droplet to be maintained. Finally, the droplets containing the desired cells are sorted by fluorescence-activated droplet sorting (FADS) [42,43]. Monoclonal antibodies are now the fastest-growing class of novel medications as a result of this application. Therefore, encapsulating individual CAR-T cells into gel droplets and then screening them can help to accelerate CAR-T development.

## 4. Microfluidic Cell Expansion for ACT

The most common and successfully developed approach to in vitro immune cell expansion is the use of magnetic beads coated with anti-CD3ε and anti-CD28 antibodies to provide costimulation and produce a high number of phenotypically low differentiated T cells [44]. However, the environment in which these signals are delivered does not precisely mirror how antigen-presenting cells (APCs) present in vivo. This may result in poor T cell proliferation rates and limited or dysfunctional T cell products. In addition, these beads are non-degradable and must be separated from the cell product prior to infusion, which may increase costs and manufacturing challenges [45,46].

Lymphocyte-based ACTs such as CAR-T cells, TCR-T cells, and TILs have demanding growth conditions and are sensitive to mechanical stress. Therefore, it is essential to construct an appropriate suspension culture using a three-dimensional bioreactor [11,47]. The three-dimensional culture of cells requires two basic elements for spherical cell aggregates and scaffolds [48]. DMSs that encapsulate single or small numbers of cells in hydrogel droplets for three-dimensional culture (cell-loaded microgels) have attracted much attention [22,49]. DMSs are well-suited to the two main foundations of three-dimensionally cultured cells. Spheroids are spherical cell aggregates based on the microspatial structure of spherical droplets, allowing seeded cells to grow and assemble into spherical clusters in a restricted environment. Scaffolds make use of materials such as natural hydrogels or synthetic gels and fibers. These materials have the appropriate porosity, permeability, mechanical properties, and surface chemistry to generate three-dimensional networks that mimic specific tissue microenvironments and allow cells to adhere and grow to form three-dimensional structures.

Additionally, DMSs reduce the facility space requirements for three-dimensional cell culture and offer several other advantages. Firstly, the reduced volume increases the surface area to volume ratio, which enhances the diffusion and exchange of small molecules and raises the sensitivity of research on cellular metabolic processes. Secondly, when constructing consistent cell culture models on a large scale, controlling compartment volume and cell numbers is beneficial for the rapid proliferation of low-differentiated cells [50]. Thirdly, the cell-loaded microgel spheres not only distribute the cells equally, but also enhance cell density. Numerous studies have demonstrated that a specified minimum cell seed density of over one million cells per cubic centimeter is necessary to rapidly activate cellular proliferation [51,52]. The DMS operational range satisfies these conditions by allowing the diameter of the microgel spheres to be made less than 50 μm. Finally, DMSs are suitable for most cellular products, easy to make, inexpensive, require no complicated handling procedures, and have good reliability.

**Figure 2:**
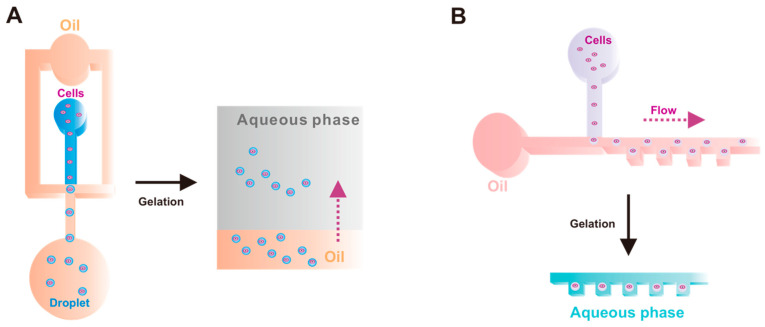
Droplet microfluidic systems. (**A**) Droplet culture. (**B**) Microtrap culture.

Currently DMSs are divided into two main categories according to the way cells are cultured: droplet culture and microtrap culture. The location of cell proliferation after gel droplet production is the primary distinction between the two techniques. In the former, the droplets are gelled, separated from the oil phase, and placed in a culture dish in the shape of spherical droplets (Figure 2A) [53]. The latter creates micro-traps in the DMS to enable the capture of droplets. Gelation and subsequent cell proliferation take place in the micro-traps (Figure 2B) [54]. The advantage of the former is that the droplets can be transported directly back into the host after the cells are proliferated and can act as a protective transport. However, there is massive droplet breakage when oil phase separation is carried out, whereas the latter does not suffer these losses. There is no additional manipulation necessary for the microtrap culture other than the flow of the aqueous phase into the channel. Therefore, the simple gelation and separation of the oil phase is an advantage of microtrap cultures. However, removing the intact droplets from the microtrap is challenging. Sart et al. [49] demonstrated that the gel in the microtrap can be melted by local light-induced heating, thus permitting the release of cells for downstream processing and allowing the DMS to better serve the ACT.

When applying a DMS to ACT, it is important to consider that the distribution of cells in the droplet will follow a Poisson distribution statistic. This is because the cell encapsulation will only contain one cell in every 10–20 drops, and the rest will be empty. In general, cell cultures do not require single-cell encapsulation. Instead, cells that can communicate with each other are grouped for faster expansion rates. Increasing the concentration of cells is one way to solve this problem, but some immune cells are difficult to obtain on a large scale. It is also possible to add a sorting step to solve this problem. Lienemann et al. [53] developed a related strategy that cleverly circumvents the limitations of the Poisson distribution by ensuring that only the droplets containing the cells can solidify to produce microgel droplets by connecting cross-linked precursors to the cells. Droplets not containing cells can then be readily removed. Without requiring a sorting step, this technique significantly increases the number of individual cell-loaded microgels that may be pooled.

## 5. Integrated Microfluidic Chips for ACT

Microfluidic technology has the advantages of automation and integration. As a result, in ACT, highly integrated microfluidic chips are created in accordance with the specifications of the final cell product. Immune cells can be processed in vitro in a flexible and minimally invasive manner, which makes it possible to prepare anti-tumor cell products quickly, easily, and affordably. The advantages of the microfluidic chip in automating the entire analytical process are fully exploited in ACT, and will propel the development of personalized ACT.

### 5.1. Integrated Chip for Cell Manipulation

Integration is one of the advantages of microfluidic chips. Microfluidic chips integrate complex cell manipulation steps such as cell separation, cell capture, and cell incubation into a single chip. It is better to manipulate the cells as required. Kim et al. designed and fabricated a high-throughput fabrication of a monodisperse cell-loaded microgel droplet microfluidic platform that can be used to prepare droplets by flowing, focusing, and separating them from the continuous oil phase through the array. This integrated chip avoids the loss of droplets in the oil phase separation step [55]. Wu et al. used microfluidic arrays and DEP to achieve parallel cell sorting and high-throughput single cell capture [56]. Karabacak et al. integrated deterministic lateral displacement, inertial focusing, and magnetophoretic sorting into a single chip to enable the separation and analysis of CTC cells [16].

Microfluidics can integrate the various steps of cell manipulation into a single micron-level chip, which greatly simplifies the manipulation steps and reduces fabrication costs. It is important to improve the clinical application of ACT strategies.

### 5.2. Integrated Chips for ACT

**Figure 3:**
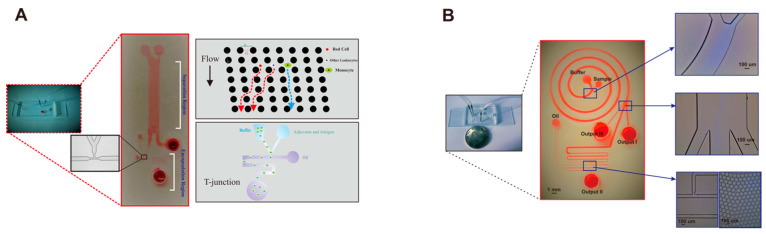
Integrated microfluidic chip for ACT. (**A**) Personalized gel-droplet monocyte vaccines for cancer immunotherapy. Reproduced with permission from Zhang et al., Lab Chip 21(22), 4414–4426 (2021). Copyright 2021 The Royal Society of Chemistry [26]. (**B**) Non-invasive T cell-adoptive immunotherapy for solid tumor with gel anti-tumor T cell injections. Reproduced with permission from Zhang et al., Chemical Engineering Journal 439, 135839 (2022). Copyright 2022 Elsevier [57].

The design and manufacture of microfluidic chips should take into account a number of factors in order to accomplish the aforementioned goals. First, it is crucial to choose effective methods for manipulating cells while minimizing the influence on cell activity and functionality, as mentioned earlier in this text. Secondly, the device should be as straightforward as possible in order to reduce the number of operational steps and the cost of the treatment. Finally, microfluidic chips for ACT are generally made up of several modules with different functions, and each module usually achieves a function similar to that of a laboratory instrument. Each module has a different functional task and is designed with different basic principles, so it is necessary to integrate the metric parameters of the different modules in order to optimize the functionality of each module as much as possible while minimizing interference between the different technologies in each module. Our group [56] has combined microfluidic deterministic lateral shift (DLD) technology with droplet microfluidics to construct an integrated chip for the isolation and culture of specific blood cells. We used the chip to make personalized gel droplet mononuclear cell vaccines for tumor therapy (Figure 3A). The DLD technology efficiently and conveniently separated monocytes from host blood and subsequently encapsulated them in gel droplets. The separation process was relatively easy, reducing the need to handle blood products and yielding highly pure monocytes. The domain-limiting effect and slow release of proteins from gel droplets were exploited to increase the efficiency of antigen uptake and antigen delivery by monocytes. Gel droplets increased the immune tolerance and the ability to target sites of action of monocyte vaccines, inducing more intense cytotoxic T lymphocyte (CTL) responses. Another microfluidic chip-based ACT strategy has also been proposed [57]. This strategy is based on microfluidic chip spiral inertial cell separation and gel droplet individual cell proliferation technology to prepare an anti-tumor T cell formulation, enabling a non-invasive T cell (NIT)-adoptive immunotherapy and the treatment of solid tumors (Figure 3B).

## 6. Summary and Future Perspectives

ACT is a personalized immunotherapy approach that has been developed rapidly in recent years. TIL for melanoma and CAR-T for malignant hematological diseases have already achieved great success. However, further optimization of this promising treatment modality is necessary. Microfluidics, with its unique advantages for processing cells, can further optimize and improve the production steps of this therapeutic modality for manufacturing cellular products. In this paper, several common microfluidic separation systems were presented. Firstly, their physical principles and applications in biological and clinical cases were described, with a focus on the ACT area. Secondly, two types of droplet microfluidic systems for culturing cells were analyzed. Droplets allow for better control of cell interactions, protect encapsulated cells from external stresses, and provide a growth environment closer to in vivo conditions. Most importantly, the encapsulated cells can be precisely and diversely manipulated, including cell separation, culture, and analysis. Finally, cell isolation and cell culture—the two crucial processes in creating ACT cell products—are integrated into a microfluidic chip, exploiting the advantages of microfluidic chip integration.

Low-cost, highly accurate, and individualized cancer treatments are possible through the use of microfluidic chips to create platforms for the manufacturing of anti-tumor cell products. With the help of microfluidics, a personalized biomedical preparation system can be created that combines cell separation, analysis, and simulation of the in vivo environment, which is essential for the large-scale cell multiplication required for therapeutic purposes. It is also necessary to combine several novel techniques [58] (such as digital microfluidics, three-dimensional printing, and artificial intelligence approaches) to create multifunctional microfluidic chips that produce reproducible, high-throughput, high-precision microfluidic devices for biomedical research. As a result, the study of biomedicine is significantly assisted by this new technology. However, it should be noted that the academic distance between developers (microfluidic engineers) and users (biologists and clinicians) hinders the transition of microfluidic systems to medicinal practices. To reduce this distance, biologists must understand the limitations of current conventional tools and provide more specific information about the purposes of medical therapies. At the same time, engineers should design innovative, user-friendly, and uncomplicated tools. In conclusion, biomedical scientists, chemists, electrical engineers, software engineers, and other specialists in relevant domains have worked together to overcome the difficulties of fundamental research and application to create and produce an artificially intelligent microfluidic chip that integrates assessment, preparation, and therapy. As a result, this chip will help ACT to become one of the best instruments in the field of cancer treatment.

## Figures and Tables

**Table 1:** Summary of cell isolation techniques.

Isolation Technique	Advantages in ACT	Limitations in ACT
Flow cytometry	Higher purity and recovery	Requires fluorescent markers and fluorescent dyes
Immunomagnetic bead sorting	High specificity and low cell damage	Requires immunomagnetic beads
Density gradient centrifugation	Common and inexpensive	Some immune cells have a specific gravity similar to lymphocytes
Cell separation based on various adhesion qualities	Low operational requirements	Most immune cells are suspended
Microfluidic technology	Easy operation, low cost, and high separation efficiency	Additional equipment is required to improve the specificity and purity of the separation

## Data Availability

Not applicable.
